# Ptychographic reconstructions performed in real time and offline have equivalent quality

**DOI:** 10.1038/s41598-025-99740-z

**Published:** 2025-04-26

**Authors:** Rebecka Lexelius, Maik Kahnt, Filipe R. N. C. Maia

**Affiliations:** 1 Laboratory of Molecular Biophysics, Department of Cell and Molecular Biology, Uppsala University, Husargatan 3 (Box 596), 75124 Uppsala, Sweden; 2 MAX IV Laboratory, Lund University, 22100 Lund, Sweden; 3 NERSC, Lawrence Berkeley National Laboratory, Berkeley, CA 94720 USA

**Keywords:** Imaging techniques, Microscopy, Computational biophysics, Nanoscale materials, Characterization and analytical techniques, Imaging techniques, Microscopy, Super-resolution microscopy

## Abstract

Ptychography is a burgeoning imaging technique that enables high-resolution, lensless reconstruction of complex samples by analysing overlapping diffraction patterns, making it invaluable in fields like materials science, biology, and nanotechnology. Real-time ptychographic reconstructions are gaining interest in the scientific community as they provide immediate feedback. Yet their potential to replace offline reconstructions remains uncertain, in part due to questions about the quality of the resulting images. This study quantitatively compares real-time and offline reconstructions at different overlap conditions. Offline reconstructions, using all diffraction patterns at once, and real-time reconstructions, where new frames are added to the reconstructions in small chunks as the diffraction patterns are recorded, were indistinguishable and identical in reconstruction quality. These results hold consistently across all tested overlap ratios. This study represents the first quantitative analysis of real-time ptychographic reconstruction using a growing dataset, demonstrating the potential for real-time reconstructions to replace or at least complement offline reconstructions.

## Introduction

X-ray ptychography^1^ is a scanning microscopy technique in which a sample is illuminated by a coherent beam over overlapping regions and the resulting diffraction patterns are collected downstream. These patterns are processed to reconstruct both the sample, often called the object, and the illuminating beam, called the probe. Since detectors only measure the intensity of diffraction patterns, not the phase, this phase information must be recovered computationally. The overlapping regions provide redundancy, which allows one to solve the phase problem and provides a constraint for the reconstruction, which ensures that one consistent object is obtained. All commonly used reconstruction algorithms are iterative and refine initial estimates of the probe and object by alternating between forward propagation of the exit wave, adjustment to match measured patterns, and backward propagation, refining the estimates until sufficient stagnation between the iterations has been observed^1^. The technique provides structural and chemical information with a wide range of applications in material science^2–5^ and biology^6,7^ and can currently achieve a 3D resolution of up to 4 nm^8^.

Traditionally, a ptychographic reconstruction is initiated only after the scan is complete, causing delays in providing feedback on experimental settings and scan quality due to lengthy processing times. This issue has become more prominent as detectors get faster and light sources get brighter, and is expected to intensify moving forward. One way to address these challenges is to perform real-time reconstructions instead. By starting the reconstruction process before the scan is finished, the iterative reconstruction process can start on the already captured diffraction patterns. As new frames come in, they are incorporated into the reconstruction with progressively refined estimates of the object and probe from previous iterations. This real-time feedback enables faster optimisation of experimental parameters, such as scanning step size, exposure time, and distance between the focus and the sample plane, based on the observed sample features. It can also help detect potential sample drift and damage. This allows researchers to stop an ongoing measurement to replace the sample if damage is observed, or to promptly adjust experimental conditions to maintain sample integrity and data quality. Suppose the real-time reconstruction is of adequate quality to be used as the final reconstruction. In that case the decision of adjusting such parameters can be made with greater confidence and would reduce the computational resources needed to be used.

Previous reports have already been published showing that real-time ptychography is possible to perform. But to what extent it would be possible to use the real-time reconstruction as a final result is less studied. Weber et al.^9^ show that real-time reconstructions visually converge to the corresponding offline reconstructions for different scanning schemes, but do not provide any error metric for the difference between the reconstructions. Welker et al.^10^ report lower normalised mean square error (NMSE) values for their real-time reconstructions compared to their offline ones. However, the real-time reconstructions are performed on a non-growing data set and the error of the offline reconstructions is very high, with the error of the real-time reconstructions being, surprisingly, slightly lower.

Here we present a careful study of the reconstruction quality of offline and real-time reconstructions on a growing dataset. To have a ground truth to fall back to, simulated datasets were used for the reconstructions. The influence of the overlap ratio in the dataset was tested by varying the step size. We compared offline and real-time ptychographic reconstructions and show that the differences between the error of the two reconstructions are similar. Further, we show that the differences between the offline and online reconstructions are negligible.

## Methods

### Simulation of the sample and diffraction patterns

**Fig. 1:**
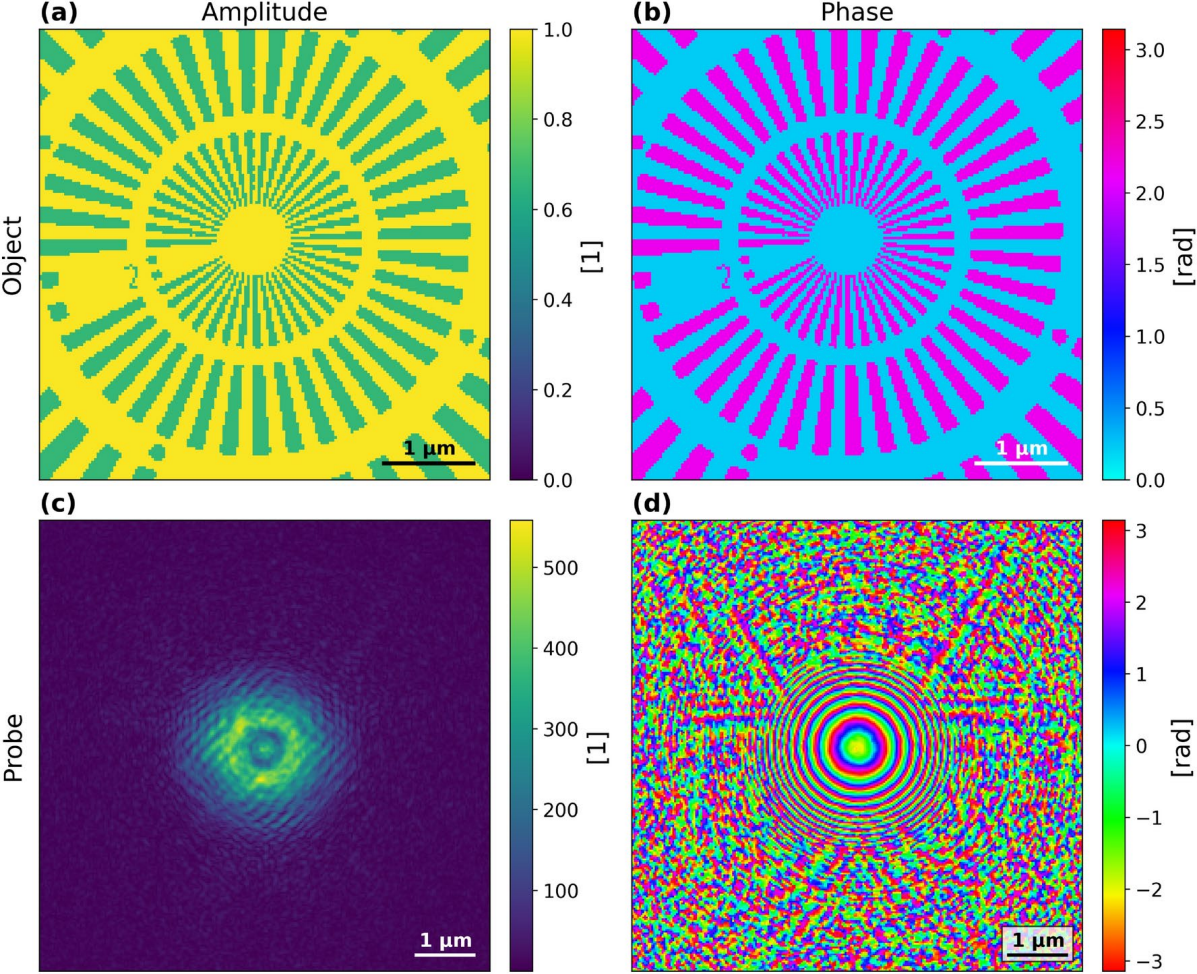
Ground truth object and probe in the top and bottom row respectively. Amplitudes are shown in the left column and phases in the right.

We modelled the sample shown in Fig. 1a,b, illustrating a $1 {\upmu \textrm{m}}$ thick gold Siemens star on a $0.5 {\upmu \textrm{m}}$ thick silicon nitride window, from the tabulated values of the complex-valued refractive indices^11^. The simulations were performed using an experimentally determined probe, from a previous reconstruction obtained at the NanoMAX beamline of MAX IV^12^, with the incident illumination intensity set to $10^{10}$ photons and energy set to 8 keV. The probe is shown in Fig. 1c,d.

Diffraction patterns of the sample with Poisson noise were simulated with PtyPy^13^. A detector with 256$\times$256 pixels (px) and pixel size of $75 {\upmu \textrm{m}}$ was used, at a distance of 3.67 m. This configuration resulted in a real-space pixel size of 29.6 nm. An Archimedean spiral scan was used starting from the centre and continuing outward. This is ideal for real-time ptychography, as incoming frames add perfectly to a concise convex, densely sampled bit of object with the overlap criterion fulfilled within the already sampled area. Each scan consisted of 315 canning points with step sizes of 10 px, 19 px, 27 px, 35 px, and 40 px, resulting in corresponding overlaps 90.0%, 80.7%, 71.0%, 60.9%, and 54.2%, calculated as described in^14^.

### Ptychographic reconstructions

The reconstructions were performed with a custom PtyPy version^15^ in single precision.

**Fig. 2:**
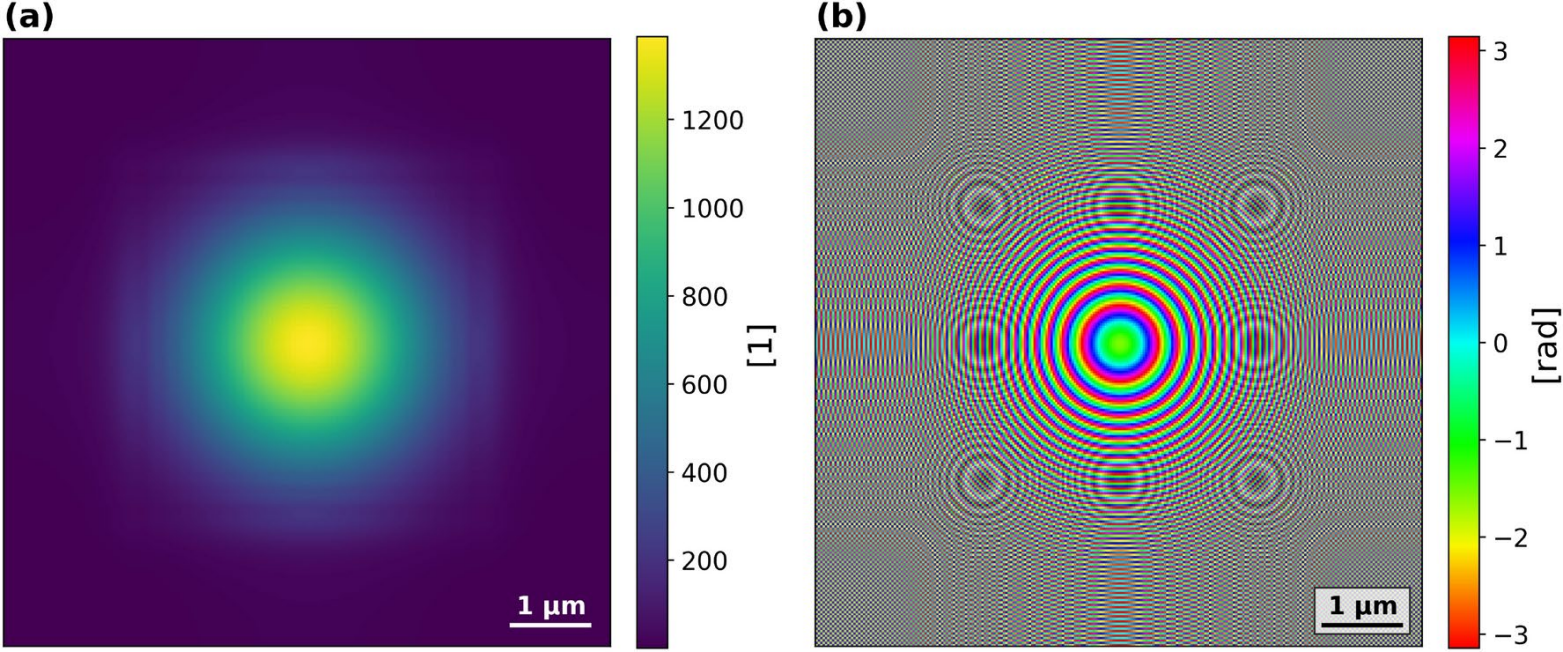
Initial probe used in all reconstructions. The amplitude is shown in (**a**) phase in (**b**).

A non-absorbing sample with a uniform phase was used as the initial object estimate, meaning that each pixel was assigned the same complex value corresponding to an amplitude of 1. The initial probe was estimated by uniformly illuminating a 50 nm wide circular aperture and then propagating the wavefront $980 {\upmu \textrm{m}}$ downstream. The initial probe is shown in Fig. 2.

For the real-time reconstructions, the position data and the diffraction frames were provided with a delay of 0.6 seconds between each data frame. This delay was chosen to match the delay from an experimental dataset obtained at NanoMAX. Data were loaded into the ptychographic engine in chunks of 25 frames and, for the real-time reconstructions, iterations were performed until the next complete chunk was available.

The same reconstruction parameters were used in all the offline- and real-time reconstructions (for details check the deposited reconstruction scripts), except for a parameter that determines after how many loaded frames the reconstruction should start. This parameter was set to 315 for the offline cases and to 1 for the real-time cases, corresponding to beginning the reconstruction after receiving the first chunk of 25 frames.

**Fig. 3:**
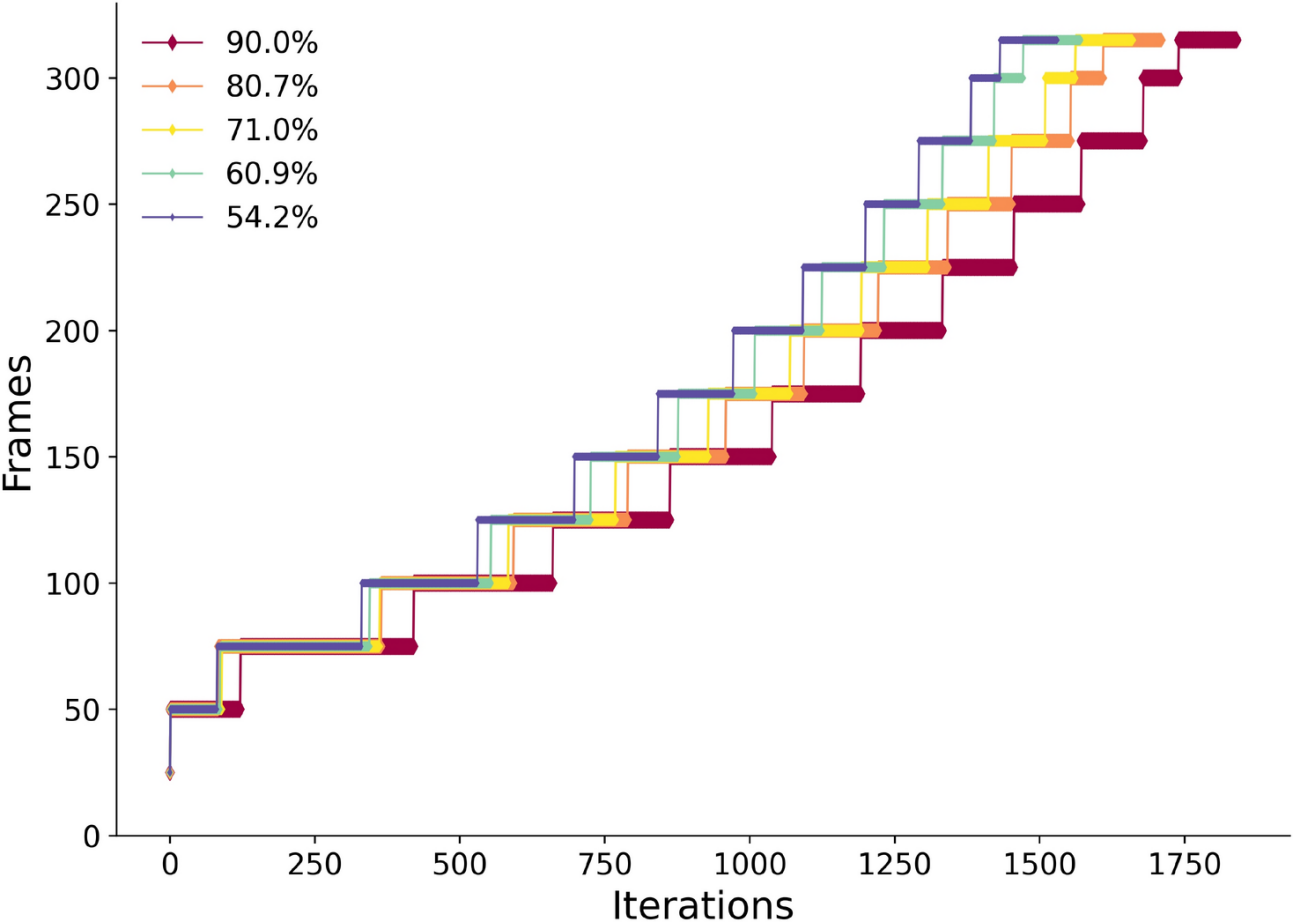
Number of data frames loaded at every iteration for all overlap reconstructions performed in real time. The percentages in the legend represent the overlap ratios. Note that the reconstruction receives at constant time intervals so the total runtime is approximately constant for the different overlaps.

The number of data frames available at each iteration, for different overlaps, is shown in Fig. 3. A mix between Difference Map (80%) and Error Reduction (ER) (20%) was used.

### Analysis

After all frames had been loaded for each real-time reconstruction, an additional 100 iterations were executed, rounded to the nearest ten. The same number of total iteration counts was also used for the corresponding offline reconstructions when evaluating the differences in the optical density (OD) and phase between the ground truth and the reconstructions.

For each overlap scenario, all three object arrays (ground truth, offline and real-time reconstructions) were cropped to 165 px $\times$ 165 px to exclude the noisy area on the edges of the reconstructed objects. The crop size was determined from the highest overlap reconstructions since these cover the smallest region of the ground truth object. Supplementary Figures S1 and S2 show the sum of the illumination beam at every scanning point together with the ground truth object and a 165 px $\times$ 165 px square indicating the cropped region. To remove the phase ramp that occurs in the reconstructions we applied PtyPy’s rmphaseramp function to all three arrays multiple times. Due to the lack of centrosymmetry in the sample, the algorithm will impose an artificial gradient on the objects. It is therefore necessary to apply the ramp removal to the ground truth as well. This ensures that the artificial gradient cancels out when calculating the differences. Potential lateral shifts between the ground truth object and the reconstructed objects were calculated using the phase_cross_correlation^16–18^ function from the module registration of the python package skimage^19^.

The reconstructed objects, $O_{r}$, are then scaled to the ground truth, $O_{g}$, by:

1 $$\begin{aligned} O_{r} = O_{r} \times \dfrac{1}{N^{2}}\sum _{i}^{N}\sum _{j}^{N} \dfrac{|O_{g}(i,j)|}{|O_{r}(i,j)|} \end{aligned}$$

where the subscript *r* denotes an output of either the offline or real-time reconstruction and *i*, *j* are the pixel indices of the cropped object arrays with shape (*N*, *N*).

The amplitudes were converted to optical densities before calculating the difference, $\epsilon _{\text {OD}}$:

2 $$\begin{aligned} \epsilon _{\text {OD}} = \log |O_{r}| - \log |O_{g}| \end{aligned}$$

To calculate the phase difference, $\epsilon _{\phi , r}$, the relative phase shift, $\Delta \phi$, was first subtracted from each reconstructed object. The relative phase shift was also calculated using the phase_cross_correlation function. The phase differences were then wrapped to be confined within the interval $[-\pi , \pi ]$,

3a $$\begin{aligned} \epsilon _{\phi , r}&=\arg \left( O_{r} \right) - \Delta \phi - \arg \left( O_{g} \right) , \end{aligned}$$

3b $$\begin{aligned} \epsilon _{\phi , r}&= {\left\{ \begin{array}{ll} \epsilon _{\phi , r} + 2\pi & \text {where } \epsilon _{\phi , r} < -\pi \\ \epsilon _{\phi , r} - 2\pi & \text {where } \epsilon _{\phi , r} > \pi \end{array}\right. } \end{aligned}$$

In addition, for comparison to previous studies, the normalised mean square error (NMSE)^20^ was calculated as,

4 $$\begin{aligned} \text {NMSE} = \dfrac{ \sum _{i}^{N}\sum _{j}^{N} |O_{g}(i,j) - \gamma O_{r}(i,j)|^{2} }{ \sum _{i}^{N}\sum _{j}^{N} |O_{g}(i,j)|^{2} }, \qquad \gamma = \dfrac{ \sum _{i}^{N}\sum _{j}^{N} O_{g}(i,j)O_{r}(i,j)^{*} }{ \sum _{i}^{N}\sum _{j}^{N} |O_{r}(i,j)|^{2} } \end{aligned}$$

where $\gamma$ enables scaling of $O_{r}$ by a constant factor and corrects for the relative phase shift between $O_{g}$ and $O_{r}$.

The corresponding differences and NMSE were also calculated between the offline and real-time reconstructions using the same equations as above, but by using the real-time reconstructed object in places where $O_{r}$ was used and the offline reconstructed object where $O_{g}$ was used.

## Results

**Fig. 4:**
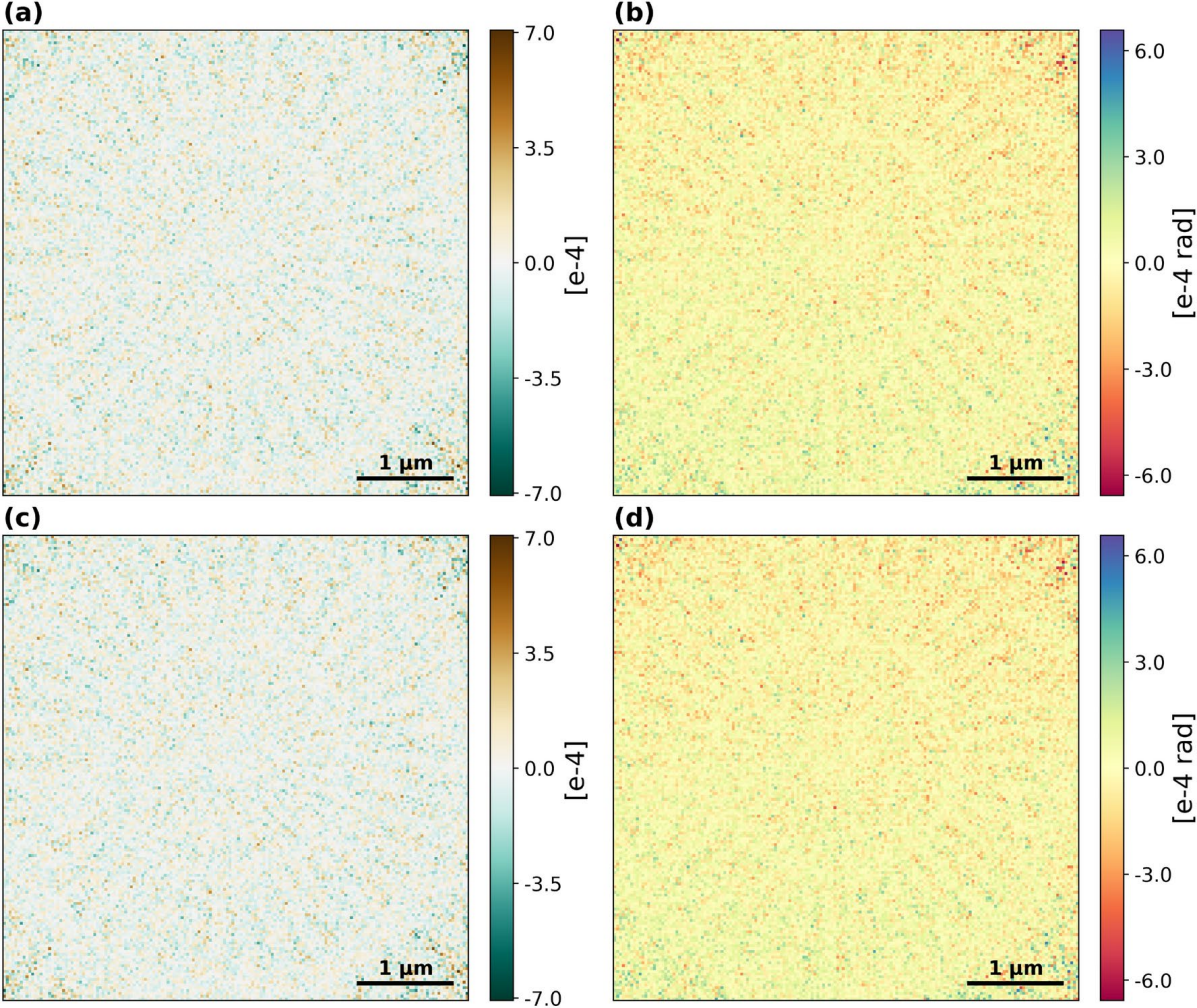
Differences between the highest overlap reconstructions to the ground truth object. (**a**) OD difference between the ground truth and offline reconstruction. (**b**) Phase difference between the ground truth and offline reconstruction. (**c**) OD difference between the ground truth and real-time reconstruction. (**d**) Phase difference between the ground truth and real-time reconstruction.

**Fig. 5:**
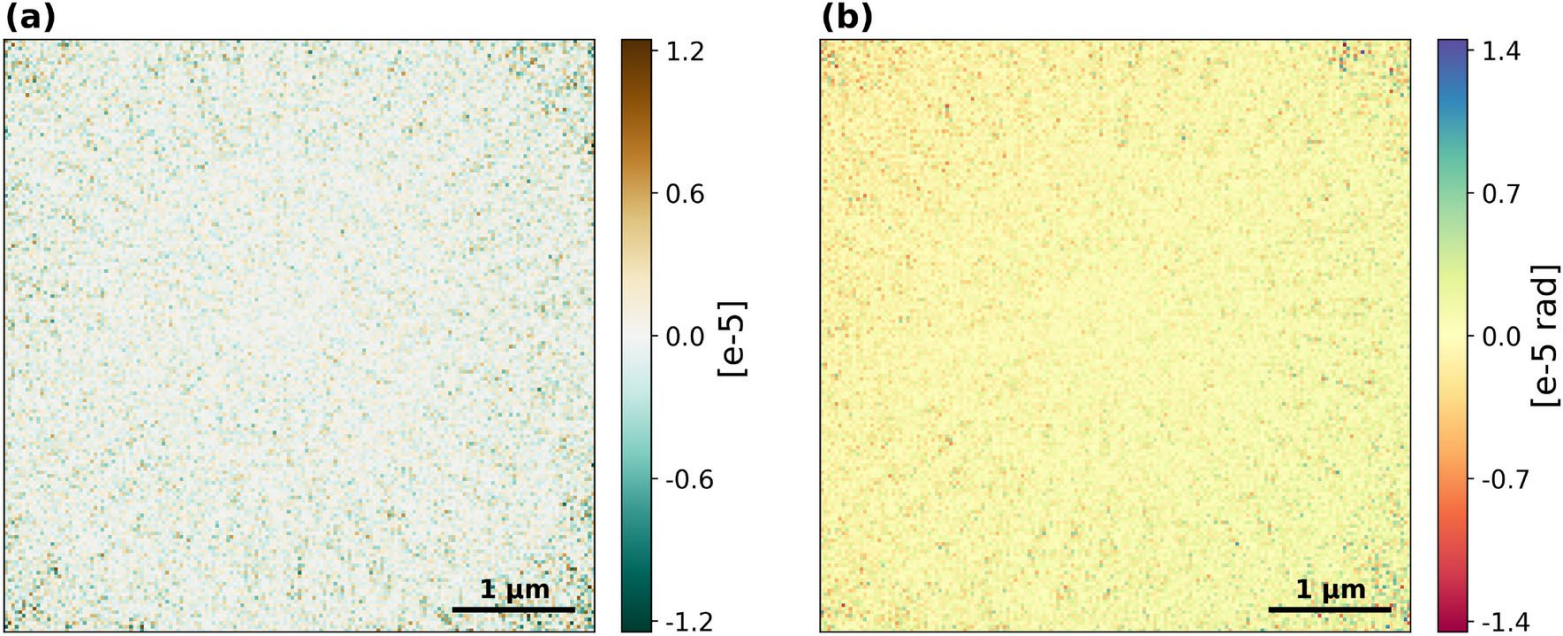
Difference between the offline reconstruction and the real-time reconstruction for the highest overlap case. (**a**) OD difference. (**b**) Phase difference.

We compared ground truth, offline and real-time reconstructions using the OD difference, $\epsilon _{\text {OD}}$, and phase difference $\epsilon _{\phi }$. The results are shown in Figs. 4 and 5 for the reconstructions with the highest overlap.

**Table 1:** Error metrics were evaluated for the highest overlap, 90.0%, and lowest overlap, 54.2%, in the top and bottom sections, respectively. In each overlap section, the errors are calculated for the offline reconstruction against the ground truth in the top row, the real-time reconstruction against the ground truth in the middle row, and the real-time reconstruction against the offline reconstruction in the bottom row. Mean values with standard errors of the absolute OD difference in the left column and of the absolute phase difference in the middle column. The right column contains the normalised mean square error (NMSE). The calculations were made on the 165$\times$165 px cropped objects. An equivalent table for the intermediate overlap simulations can be found in Supplementary Table S1.

		OD difference, $\epsilon _{\text {OD}}$	phase difference, $\epsilon _{\phi }$	NMSE
High overlap	Offline - Ground truth	7.81e-05 ± 3.89e-07	7.99e-05 ± 3.99e-07	1.81e-08
	Real-time - Ground truth	7.81e-05 ± 3.89e-07	8.00e-05 ± 3.99e-07	1.82e-08
	Real-time - Offline	1.48e-06 ± 7.49e-09	1.49e-06 ± 7.62e-09	6.56e-12
Low overlap	Offline - Ground truth	3.10e-04 ± 1.56e-06	3.72e-04 ± 1.77e-06	3.41e-07
	Real-time - Ground truth	2.99e-04 ± 1.51e-06	3.98e-04 ± 1.83e-06	3.60e-07
	Real-time - Offline	8.05e-05 ± 4.12e-07	1.09e-04 ± 5.18e-07	2.73e-08

The mean values of the absolute value of the OD and phase differences for each pair of objects are listed in the left and middle column of Table 1, respectively, to enable a quantitative comparison. The NMSE, calculated using equation 4, are listed in the right column. Additionally, the NMSE was calculated for all object pairs in the lowest overlap reconstruction at every 10th iteration (see Supplementary Fig. 3).

Phase ramps were removed before all calculations were performed. No alignment was needed for the reconstructed objects at the final iterations. However, alignment was necessary for the objects evaluated at every 10th iteration during earlier stages of the reconstruction. Scaling of the amplitudes and subtraction of the relative phase shift were also applied before calculating the differences and NMSE.

With these corrections applied, we found no substantial difference in the error metrics of the offline and the real-time reconstructions. The differences between the two types of reconstructions were always smaller than the difference with the ground truth. The NMSE as a function of the number of iterations quickly converged to similar values for the offline and real-time reconstructions.

## Discussion

This study investigates the potential of real-time ptychographic reconstructions to achieve a level of quality comparable to that of offline methods. To facilitate a rigorous comparison, we employed an artificial sample as the ground truth, thereby eliminating potential experimental artifacts and enabling a more precise identification of discrepancies between reconstruction techniques. This approach allows us to directly contrast the differences between real-time and offline reconstructions with the individual deviations of each from the known ground truth. Crucially, we found that the disparity between the offline and real-time reconstructions was consistently smaller than the difference between either reconstruction and the ground truth, a finding that held across all overlap ratios examined. Consequently, evaluating the difference between real-time and offline reconstructions emerges as a valuable proxy for assessing the accuracy of the real-time approach, particularly relevant in experimental settings where the true object is unknown.

Previous work by Weber et al.^9^ demonstrated that real-time ptychography could yield visually similar results to offline reconstructions using the ePIE algorithm^20^ on experimentally acquired data with varying scanning schemes. However, their analysis lacked quantitative comparisons, and artifacts were evident in the difference images. Furthermore, their study aimed to assess the suitability of real-time reconstructions as a preview tool rather than a direct replacement for offline processing. In contrast, by correcting for inherent ptychographic ambiguities—specifically constant phase shifts, arbitrary object scaling, lateral shifts, and phase ramps —we effectively eliminated the artifacts observed in their difference images. This refinement provides a more accurate measure of the true divergence between offline and real-time reconstructions, resulting in demonstrably lower errors.

Conversely, Welker et al.^10^ reported lower normalised mean squared error (NMSE) values against the ground truth for real-time reconstructions compared to offline ones, a finding we were unable to replicate in this study. Their real-time algorithm employed a strategy of updating the reconstruction using fixed-size subsets of frames, effectively freezing portions of the object after a predetermined number of iterations. While we utilised a similar scanning pattern, overlap ratios, reconstruction algorithms, and error metric, the errors reported in our study are approximately six orders of magnitude lower than those reported by Welker et al. This substantial reduction in error persists even when examining our real-time reconstruction results after 100 iterations, mirroring their evaluation point.

Our findings strongly suggest that, within the tested range of overlap ratios, real-time ptychographic reconstructions can indeed serve as a viable replacement for offline methods without compromising image quality. This conclusion implies that the overlap ratio, at least within the parameters explored here, is not a fundamental limitation for achieving accurate real-time reconstructions. The ability to perform ptychographic reconstructions in real time with comparable quality to offline methods offers significant advantages beyond mere monitoring. Reliable real-time results empower researchers to make more informed decisions regarding experimental adjustments during ongoing scans. For instance, the ability to confidently assess data quality in real time allows for dynamic adjustments, such as reducing the step size for denser datasets, thereby optimising data collection and storage. Eliminating the need for post-experiment offline processing also accelerates the analysis pipeline, allowing researchers to analyse results sooner and utilise computational resources more efficiently. This ultimately reduces the time from data acquisition to publication.

Although this study employed simulations of an artificial sample, the resulting reconstructions are expected to closely mirror those from real experiments. The primary distinction lies in the use of precisely known probe positions in our simulations, whereas real-world experiments have minor errors in recorded positions compared to the true values. For real-time reconstructions to succeed it is also important that the scan pattern provides sufficient overlap between the recorded frames throughout the reconstruction, as is the case for the spiral scans used here. While this study focused on the impact of overlap ratio, other factors may influence real-time reconstruction performance and warrant future investigation. These include, for example, the distribution of iterations between loading new frames, the chunk size of loaded frames, the number of probe modes utilised to handle partial coherence, photon noise and other noise sources.

## Conclusions

This study demonstrates the viability of real-time ptychography as a direct replacement for offline processing, achieving comparable image quality within the tested overlap ratio range. The consistent finding that the disparity between real-time and offline reconstructions is smaller than their individual deviations from the ground truth underscores the robustness of the real-time approach and its potential for accurate, on-the-fly data assessment. This capability moves beyond simple previewing, offering researchers the power to dynamically optimise experiments, reduce data storage needs through informed adjustments, and drastically accelerate the time to results. While simulations provided a controlled environment for this investigation, the insights gained are highly relevant to experimental practice. Future work should now focus on investigating the impact of factors beyond overlap ratio, including iteration strategies, data chunking, and advanced reconstruction parameters, to fully realise the potential of real-time ptychography in diverse experimental contexts.

## Supplementary Information

Supplementary Information.

## Data Availability

Data and software scripts are available from https://doi.org/10.5281/zenodo.14673903.
