# Supplementary material for: Ptychographic reconstructions performed in real time and offline have equivalent quality
Source: Sci Rep. 2025 Apr 26;15:14674. doi: 10.1038/s41598-025-99740-z (PMC12033293; doi:10.1038/s41598-025-99740-z)
Supplement: Supplementary file 1 — Supplementary Information. [file 41598_2025_99740_MOESM1_ESM.pdf]

Ptychographic reconstructions performed in real time and offline have equivalent quality

Supplementary Information

Rebecka Lexelius[1], Maik Kahnt[2,*], and Filipe R. N. C. Maia[1,3,*]

[1]Laboratory of Molecular Biophysics, Department of Cell and Molecular Biology, Uppsala University, Husargatan 3 (Box 596), SE-75124, Uppsala, Sweden
[2]MAX IV Laboratory, Lund University, 22100 Lund, Sweden
[3]NERSC, Lawrence Berkeley National Laboratory, Berkeley, CA, 94720, USA
[*]maik.kahnt@maxiv.lu.se,filipe.maia@icm.uu.se

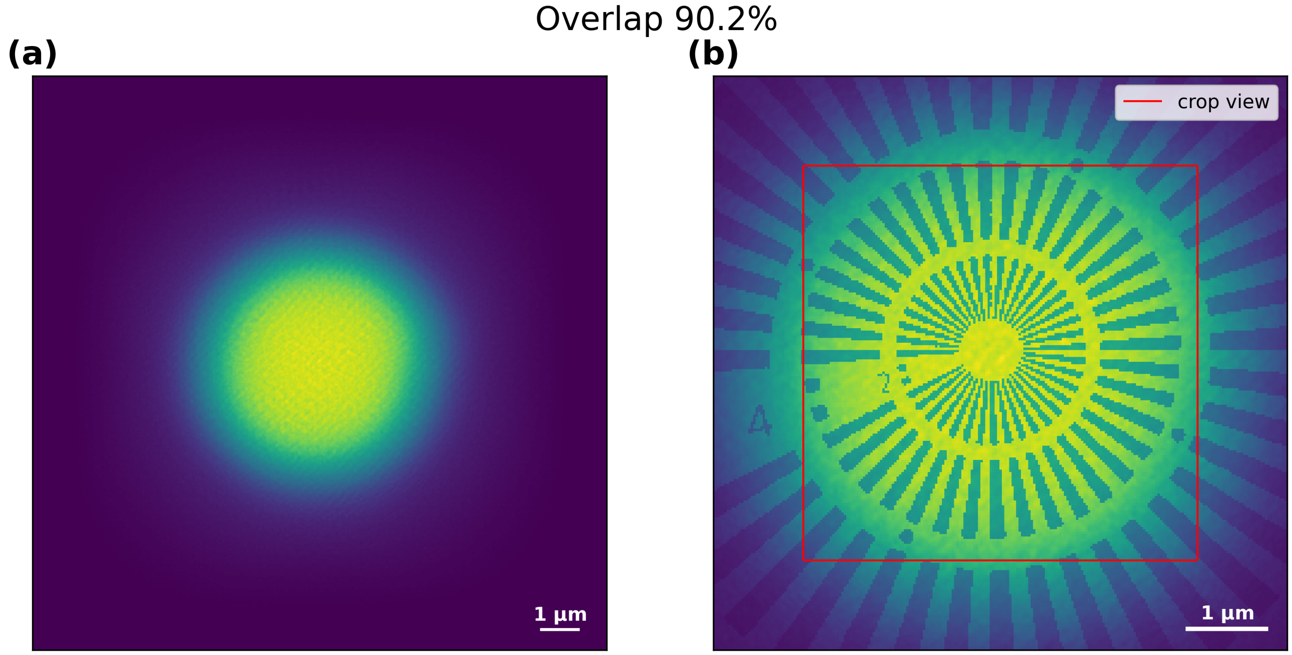

Figure S1. Total illumination area for the highest overlap scan. (a) Summed illuminated area using the amplitude of the ground truth probe at scanning positions used for the highest overlap reconstructions. (b) Illuminated area projected on the ground truth object.

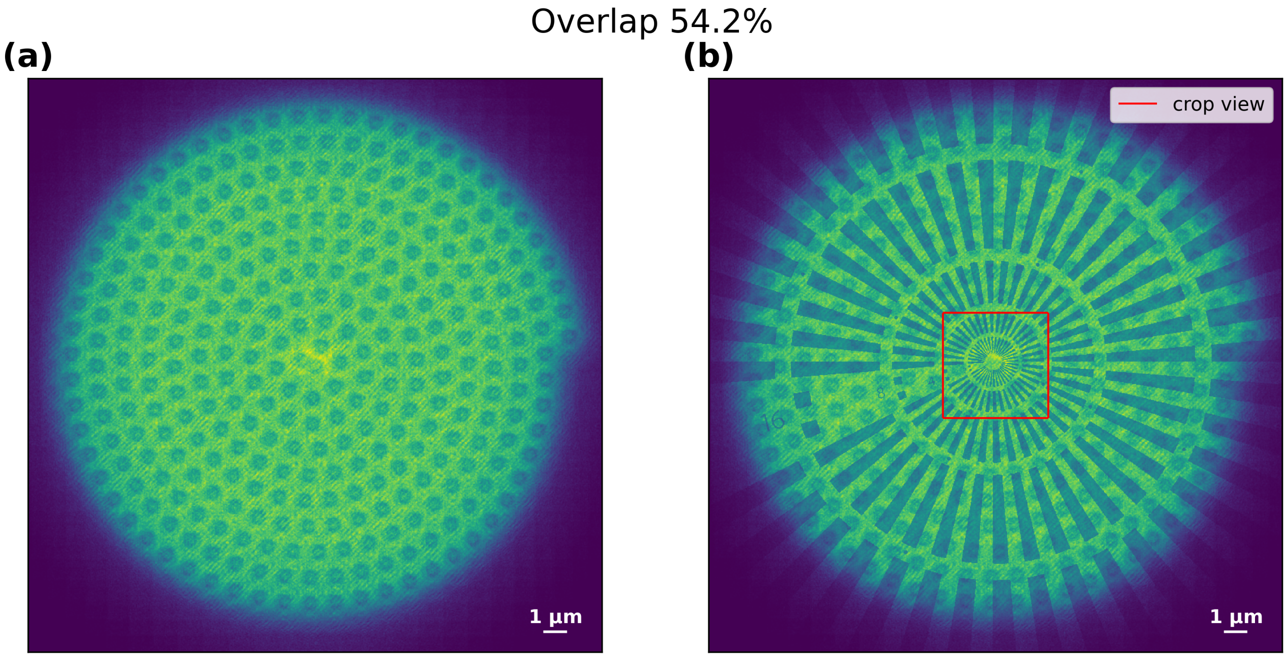

Figure S2. Total illumination area for the lowest overlap scan. (a) Summed illuminated area using the amplitude of the ground truth probe at scanning positions used for the lowest overlap reconstructions. (b) Illuminated area projected on the ground truth object.

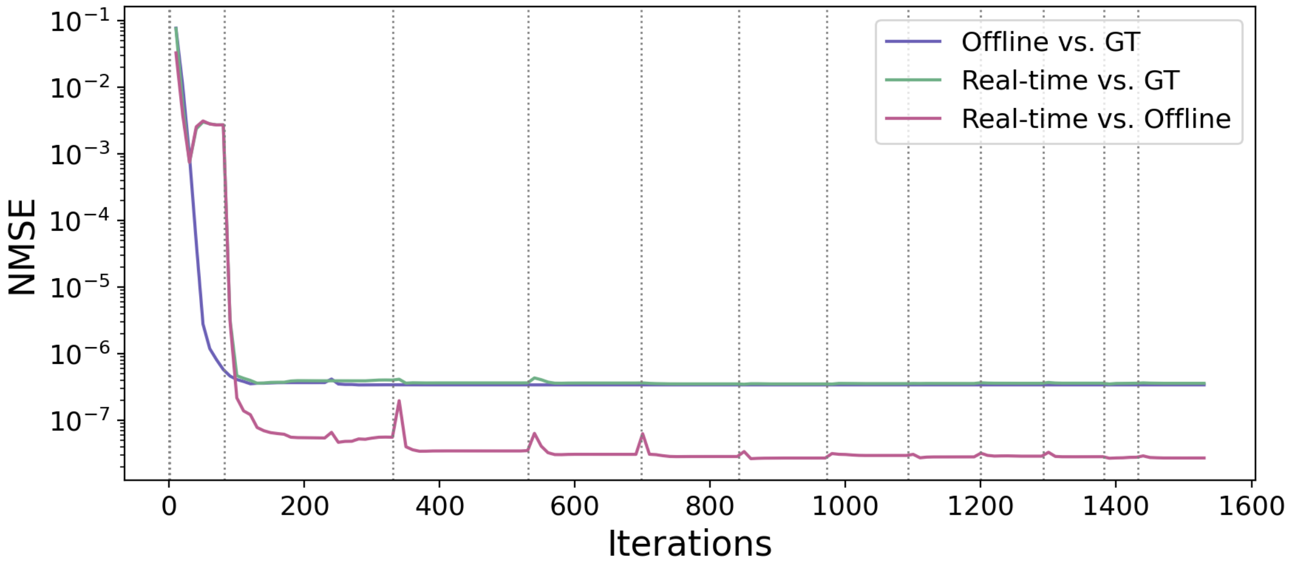

Figure S3. Normalised mean square error as a function of number of iterations performed, on the lowest overlap reconstructions. Evaluated at every 10th iteration on the central 165 px × 165 px region. The grey dotted lines indicate where new frames were added to the real-time reconstruction.

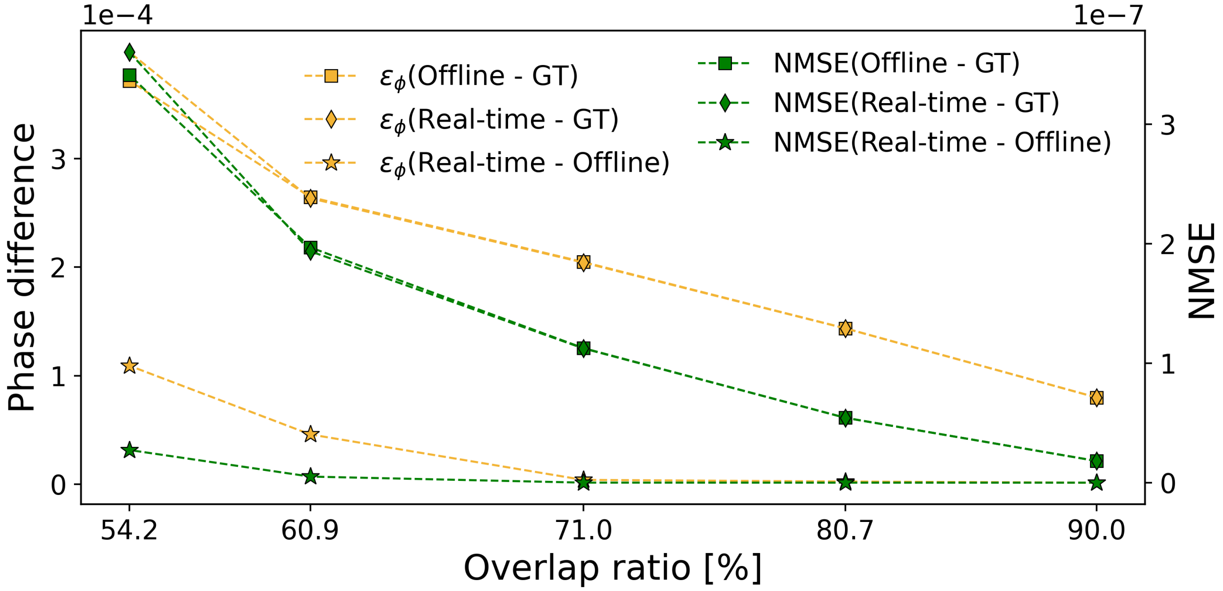

Figure S4. Error metrics as a function of overlap ratio. Yellow: mean value of the absolute phase difference, green: normalised mean square error. Values have been evaluated on the central 165 px × 165 px region.

Overlap	Comparing cases	OD difference, ε_{OD}	phase difference, ε_ϕ	NMSE
90.0%	Offline - Ground truth	7.81e-05 ± 3.89e-07	7.99e-05 ± 3.99e-07	1.81e-08
	Real-time - Ground truth	7.81e-05 ± 3.89e-07	8.00e-05 ± 3.99e-07	1.82e-08
	Real-time - Offline	1.48e-06 ± 7.49e-09	1.49e-06 ± 7.62e-09	6.56e-12
80.7%	Offline - Ground truth	1.33e-04 ± 6.39e-07	1.44e-04 ± 6.81e-07	5.43e-08
	Real-time - Ground truth	1.33e-04 ± 6.39e-07	1.44e-04 ± 6.81e-07	5.43e-08
	Real-time - Offline	2.51e-06 ± 1.23e-08	2.54e-06 ± 1.25e-08	1.83e-11
71.0%	Offline - Ground truth	1.93e-04 ± 9.26e-07	2.05e-04 ± 9.80e-07	1.13e-07
	Real-time - Ground truth	1.93e-04 ± 9.26e-07	2.04e-04 ± 9.78e-07	1.12e-07
	Real-time - Offline	3.61e-06 ± 1.79e-08	4.06e-06 ± 1.96e-08	4.29e-11
60.9%	Offline - Ground truth	2.56e-04 ± 1.27e-06	2.65e-04 ± 1.31e-06	1.97e-07
	Real-time - Ground truth	2.53e-04 ± 1.25e-06	2.63e-04 ± 1.29e-06	1.94e-07
	Real-time - Offline	3.80e-05 ± 1.89e-07	4.59e-05 ± 2.18e-07	5.09e-09
54.2%	Offline - Ground truth	3.10e-04 ± 1.56e-06	3.72e-04 ± 1.77e-06	3.41e-07
	Real-time - Ground truth	2.99e-04 ± 1.51e-06	3.98e-04 ± 1.83e-06	3.60e-07
	Real-time - Offline	8.05e-05 ± 4.12e-07	1.09e-04 ± 5.18e-07	2.73e-08

Table S1. Error metrics evaluated for all overlap cases, after being cropped to 165x165 pixels. In each overlap section, the errors are calculated for the offline reconstruction against the ground truth in the top row, the real-time reconstruction against the ground truth in the middle row, and the real-time reconstruction against the offline reconstruction in the bottom row. Mean values with standard errors of the absolute OD difference in the left data column and of the absolute phase difference in the middle data column. The right column contains the normalised mean square error (NMSE).
